# Interactions of Nitrogen Source and Rate and Weed Removal Timing Relative to Nitrogen Content in Corn and Weeds and Corn Grain Yield

**DOI:** 10.1155/2017/8961367

**Published:** 2017-04-11

**Authors:** Alexandra M. Knight, Wesley J. Everman, David L. Jordan, Ronnie W. Heiniger, T. Jot Smyth

**Affiliations:** Department of Crop and Soil Sciences, North Carolina State University, Raleigh, NC 27695, USA

## Abstract

Adequate fertility combined with effective weed management is important in maximizing corn (*Zea mays* L.) grain yield. Corn uptake of nitrogen (N) is dependent upon many factors including weed species and density and the rate and formulation of applied N fertilizer. Understanding interactions among corn, applied N, and weeds is important in developing management strategies. Field studies were conducted in North Carolina to compare corn and weed responses to urea ammonium nitrate (UAN), sulfur-coated urea (SCU), and composted poultry litter (CPL) when a mixture of Palmer amaranth (*Amaranthus palmeri* S. Wats.) and large crabgrass (*Digitaria sanguinalis* L.) was removed with herbicides at heights of 8 or 16 cm. These respective removal timings corresponded with 22 and 28 days after corn planting or V2 and V3 stages of growth, respectively. Differences in N content in above-ground biomass of corn were noted early in the season due to weed interference but did not translate into differences in corn grain yield. Interactions of N source and N rate were noted for corn grain yield but these factors did not interact with timing of weed control. These results underscore that timely implementation of control tactics regardless of N fertility management is important to protect corn grain yield.

## 1. Introduction

Fertility and weed management are often considered two of the most critical management factors impacting corn yield [1, 2]. Nitrogen (N) uptake efficiency can be influenced by tillage, soil properties, interactions among crops and weeds, and the rate and source of N applied. Noellsch et al. [3] reported that time-release fertilizers provided N to crops gradually and improved N uptake efficiency and in some instances increased yield over other fertilizer sources that were more readily available for plant uptake. Nitrogen uptake efficiency is defined as total plant N per unit of soil N [4] and has been shown to have a strong positive correlation with photosynthesis, biomass production, kernel number, and grain yield of corn [5]. Janat [6] suggested that understanding N uptake processes and N recovery within a plant enables application of N at appropriate rates and stages of crop growth. Competition for N between crops and weeds can result in reduced available N for uptake and utilization by the crop, impacting crop yield [7–9].

The uptake of N in a cropping system is based on the requirements of the crop as well as on the availability of N in soil [10]. Nitrogen use efficiency represents the combination of N uptake efficiency, N use efficiency in plant tissue, and grain N concentration at harvest [4]. Adeli et al. [11] reported that inorganic N fertilizers increased corn yield by 43% and biomass by 30% over the nontreated corn, while organic poultry litter increased yield by 14% over the nontreated corn. Greater corn yield with inorganic fertilizer corresponded with the highest nitrate level in the soil, while pelletized poultry litter treatment provided higher levels of soil nitrate compared to the nonpelletized poultry litter. Adeli et al. [11] indicated that inorganic N fertilizer caused N uptake to rise by 48% over nontreated corn with grain N utilization increasing by 57%. Nitrogen use efficiency was 56% greater when nonpelletized poultry litter was incorporated in soil rather than broadcast to the soil surface [11]. Variation in corn response was observed when inorganic N applied is in an immediately available form or in a time-release form [3, 12, 13].

Weed-crop interference is an important management consideration. Uremis et al. [14] reported 38 to 59% reduction in corn yield through interference from field bindweed (*Convolvulus arvensis* L.), purple nutsedge (*Cyperus rotundus* L.), and puncturevine (*Tribulus terrestris* L.). Fausey et al. [15] indicated that the relationship between weed species and density and yield is hyperbolic with yields decreasing steadily due to an increase in weed density until reaching a threshold value. Weed interference at early stages of corn development decreases corn yield [7, 16–19] often by reducing corn leaf area, leaf biomass, and total biomass [20]. Similar to corn response to applied N, weed growth increases with increasing N fertilization [21, 22]. Lindsey et al. [23] reported greater N content in weeds when higher rates of N were applied. Root growth increased in proportion to shoot growth when N was limited in a cropping system [21, 23].

Yield losses of corn due to weeds were similar across N rates [24, 25]. Wortman et al. [25] reported that velvetleaf (*Abutilon theophrasti* Medik.) was able to compete more effectively with corn in soils with a high mineralization potential. Lindquist et al. [19] found that when grown in an environment together weeds and crops will impact the resources available for one another and will do so differently depending on the amount of N and other resources. Blackshaw et al. [22] observed less impact of broadleaf weeds on wheat (*Triticum aestivum* L.) yield compared to grass weed species. Furthermore, Evans et al. [26] indicated that weeds will have less impact on crop yield when N is applied early in the season even at rates below those recommended for optimum yield. Application of N early in the season also led to less weed biomass than N applications occurring later in the season [27, 28]. However, regardless of timing of application, weed biomass is often greater when N is applied [28].

It is suspected that when weeds are allowed to grow to 8 or 16 cm in height, the N content in corn plants will be lower than N content in absence of weeds and that corn yield will be lower due to competition for N by weeds. Research in North Carolina and surrounding states on coastal plain soils has not addressed the interaction of N source and rate in presence of different weed management strategies. It is also suspected that corn exposed to higher N rates will result in greater N content in the plant species present and greater corn yield will be observed in those treatments with urea ammonium nitrate over the organic fertilizer or even over the time release fertilizer. Therefore, research was conducted in North Carolina to determine corn and weed response to N source and rate based on timing of weed removal within the first 28 days of the growing season.

## 2. Materials and Methods

The experiment was conducted in North Carolina during 2011 and 2012 at the Central Crops Research Station located near Clayton (35°40′6.04′′N, 78°30′7.00′′W) and the Upper Coastal Plain Research Station located near Rocky Mount (35°53′38.94′′N, 77°40′47.50′′W). Glyphosate-tolerant corn (Hybrid DKC 63-84, Monsanto, St. Louis, MO 63167) was planted in conventionally prepared, raised seedbeds in rows spaced 97 and 92 cm apart at Clayton and Rocky Mount, respectively. The in-row plant population was five plants m^−1^. Soil at Clayton during 2011 was a combination of a Rains sandy loam (fine-loamy, siliceous, semiactive, thermic* Typic Paleaquults*), Varina loamy sand (fine, kaolinitic, thermic* Plinthic Paleudults*), a Wagram loamy sand (loamy, kaolinitic, thermic* Arenic Kandiudults*), and Norfolk loamy sand (fine-loamy, kaolinitic, thermic* Typic Kandiudults*). Soil at this location during 2012 was a combination of a Rains sandy loam and Lynchburg sandy loam (fine-loamy, siliceous, semiactive, thermic* Aeric Paleaquults*). At Rocky Mount during both years soil was a combination of a Goldsboro fine sandy loam (fine-loamy, siliceous, subactive, thermic* Aquic Paleudults*) and Norfolk loamy sand. Soil pH ranged from 5.8 to 6.3 and organic matter content did not exceed 1.8%. Plot size was 9.1 m by 3.8 m in Clayton and 9.1 m by 3.7 m in Rocky Mount.

Treatments consisted of three N fertilizers, composted poultry litter (CPL), urea ammonium nitrate (UAN), and sulfur-coated urea (SCU), applied at 0, 68, 135, and 202 kg N ha^−1^. Rates for N were made with plant available N for the synthetic fertilizers and total N content for CPL. Following each preplant incorporated N treatment, weeds were removed when the average height of the weed complex was 8 cm or 16 cm using herbicides and subsequent hand removal to keep plots weed-free for the remainder of the season. These respective timings were at V2 and V3 stages of corn development [29] and corresponded to 22 and 28 days after planting. A weed-free control for each fertilizer treatment was also included. At each weed removal timing glyphosate (Roundup PowerMAX herbicide, Monsanto, St. Louis, MO 63167), atrazine (AAtrex, Syngenta, Wilmington, DE 19810), and* S*-metolachlor (Dual II Magnum, Syngenta, Wilmington, DE 19810) were applied at 0.84 kg ai ha^−1^, 2.2 kg ai ha^−1^, and 1.12 kg ai ha^−1^, respectively. This herbicide treatment was applied after planting but prior to corn emergence or at the appropriate timing for weed removal. Herbicides were applied in 145 L ha^−1^ aqueous solution using a CO_2_-pressurized backpack sprayer fitted with 8002 regular flat fan nozzles (Spraying Systems Co., Wheaton, IL 60189).

Immediately prior to weed removal, weed density and tissue from above-ground biomass of 6 corn plants and Palmer amaranth and large crabgrass from 0.25 m^−2^ were collected from each plot randomly for N analysis. Samples were ground to particles of 2 mm or less and processed for total N content using a model 2400 CHN Elemental Analyzer (PerkinElmer Corporation, Waltham, MA 02451). Data for N content for corn, Palmer amaranth, and large crabgrass were collected via a combustion process with pure oxygen [30]. Corn grain yield was determined from the center two rows of each plot and adjusted to a final yield of 15.5% moisture.

The experimental design was a randomized complete block with treatments replicated four times. Data for N content in corn, Palmer amaranth, and large crabgrass and corn grain yield were subjected to ANOVA with partitioning for the 4 (environment) × 3 (N source) × 2 or 3 (weed height at timing of weed removal) factorial arrangement of treatments using the PROC GLM procedure in SAS depending upon sampling timing [31]. The no-N control was not included in the analyses to allow consideration of the factorial treatment structure in ANOVA. Means of significant main effects and interactions were separated using Fisher's protected LSD test at *p* ≤ 0.05.

## 3. Results and Discussion

### 3.1. General Comments

A wide range of main effects and interactions were noted among treatment factors. The discussion that follows will focus on interactions of environment, N source, and N rate with weed height at timing of weed removal corresponding to 22 and 28 days after planting.

### 3.2. Density of Large Crabgrass and Palmer Amaranth

The interaction of environment (combination of year and location) × N source × average weed height at time of weed removal was significant for large crabgrass density but not for Palmer amaranth density. This interaction is most likely associated with environments, field conditions affecting corn and weed growth, and natural distribution of weeds in the field (Table 1). At Clayton during 2011 and Rocky Mount during 2012, the decrease in weed density at the 16 cm timing of weed removal was observed compared with density when weeds were removed at weed height approximately 8 cm regardless of N source. There was also no difference in density across sources when comparing within a timing of weed removal. At Rocky Mount during 2011, no difference in large crabgrass density was noted when UAN and CPL were applied when comparing timings of weed removal. However, in this environment, a decrease in density was noted as timing of weed removal was delayed when SCU was applied. Although no difference in weed density was noted during 2012 at Clayton when UAN was applied, a decrease in weed population was observed when timing of weed removal was delayed for both SCU and CPL. Additionally, higher densities at the 8 cm timing of weed removal were noted following SCU and CPL compared with UAN.

Interactions of environment × N source and environment × weed height at timing of weed removal were significant. These interactions were caused by differences in Palmer amaranth density at Clayton during 2011 when comparing among N sources and between weed heights when weeds were removed (Table 2). A higher density of Palmer amaranth was observed following CPL compared with UAN or SCU at this location. The cause of this interaction is not known. Possibilities include the impact of CPL on germination and emergence compared with UAN and SCU or presence of Palmer amaranth in CPL. However, the latter contribution is unlikely given that the same source of CPL was applied at both locations during both years. The lower density noted at Clayton during 2011 when weeds were removed at 16 cm rather than 8 cm may have been caused by interspecific competition and loss of plants by the later timing of removal compared with the earlier timing of removal. Other researches [32] has shown that weed populations decrease later in the season due to interspecific competition among plants.

Under the conditions and procedures used in this research, the interaction of N source and N rate and timing of weed removal could not be explained fully. Future research with defined and consistent weed densities most likely could further elucidate the cause of these interactions. Nonetheless, these environments were selected because weed densities were relatively uniform across the entire experimental area. Interference of Palmer amaranth and large crabgrass for N and other resources complicates interpretation of results for individual weed species.

### 3.3. Nitrogen Content in Large Crabgrass and Palmer Amaranth

**Figure 1:**
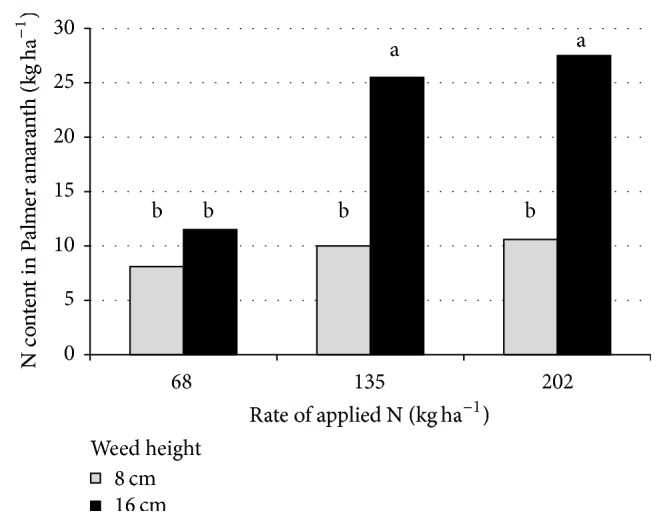
Nitrogen (N) content in Palmer amaranth plants as influenced by N rate and average weed size at timing of removal. Means followed by the same letter are not significantly different based on Fisher's protected LSD test at *p* ≤ 0.05. Data are pooled over years, locations, and N sources.

Nitrogen content in large crabgrass was affected by the interaction of environment × N source × N rate × weed height at timing of removal (Table 3). In many cases, increasing the N rate and delaying the timing of large crabgrass removal increased N content in this weed. One exception was results at Rocky Mount during 2012 where there was no difference in N content when comparing timings of weed removal. In contrast, the interaction of N rate × weed height at timing of weed removal was significant for N content in Palmer amaranth. Irrespective of N source, there was no difference in N content of Palmer amaranth when this weed was removed at a height of 8 cm (Figure 1). In contrast, when the rate of N was increased from 68 kg ha^−1^ to 135 or 202 kg ha^−1^, the content of N in above-ground Palmer amaranth biomass increased.

### 3.4. Nitrogen Content in Corn at Different Timings of Weed Removal

The interaction of environment × N source × N rate × weed removal timing was significant for N content in corn. This interaction was noted for comparisons of removal when weeds were 8 cm or 16 cm in height compared with weed-free corn. At Clayton and Rocky Mount during 2011, N content in corn was higher when SCU was applied at 202 kg/ha for weed-free corn compared with corn grown with weeds until weeds were removed at the 8 cm timing (Table 4). However, no difference in N content in corn was noted when lower rates of SCU were applied or for other sources of N regardless of rate.

When comparing N content in corn when weeds were removed at a height of 16 cm, no differences were noted during 2012 regardless of location (Table 5). However, differences were noted when comparing N content of corn in absence of weeds compared with interference for 28 days (16 cm weed removal timing) at both locations during 2011. Less N was found in corn following weed interference when both UAN and SCU were applied at 135 and 202 kg/ha or when CPL was applied at 202 kg/ha. Fewer differences were noted at Rocky Mount during 2011; however, samples for N rates of 135 and 202 kg/ha from SCU were lost during processing and limit conclusions pertaining to comparison of N sources. With respect to UAN at this location, N content in corn was lower only when less than 202 kg/ha of N was applied. Unlike results at Clayton, no difference in N content was noted among weedy and weed-free treatments when 202 kg/ha of N was applied as CPL.

### 3.5. Corn Grain Yield

**Figure 2:**
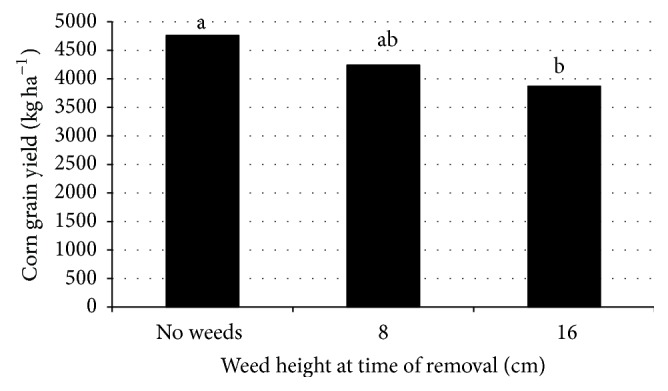
Influence of average weed height at timing of weed removal on corn grain yield. Means followed by the same letter are not significantly different based on Fisher's protected LSD test at *p* ≤ 0.05. Data are pooled over years, locations, N sources, and N rates.

Main effects of environment, N rate, and average weed height at time of weed removal were significant for corn grain yield. The interaction of environment × N source × N rate was also significant. Corn yield was greater when corn was maintained weed-free immediately after planting and for the remainder of the growing season compared with interference caused by weeds in the field until weeds reached a height of approximately 16 cm (Figure 2). The number of days between planting and application of herbicide to remove weeds at a height of 16 cm was 28 days. There was no difference in yield when weeds were removed when the average height was 8 cm compared with either the weed-free control or weed removal at weed height of 16 cm. Parker et al. [33] reported that delaying herbicide applications by one or two weeks early in the season could result in reduced grain yield from weed interference compared with corn grown in absence of weeds. This research was conducted in North Carolina in presence of Palmer amaranth, large crabgrass, and other problematic weeds.

Differences in corn grain yield varied depending on environment, N source, and N rate irrespective of when weeds were removed. When pooled over average weed height at timing of removal, corn yield did not differ regardless of N rate when comparing within CPL treatment (Table 6). This was also the case in 3 of 4 environments when UAN was applied (Clayton and Rocky Mount during 2011 and Rocky Mount during 2012). At Clayton in 2012, corn grain yield following application of N at 202 kg ha^−1^ as UAN exceeded that of corn receiving 68 kg N ha^−1^ with yield following 135 kg N ha^−1^ intermediate between these rates. No consistent trend was observed when comparing corn response to N rate when SCU was applied. At Clayton during 2011, corn yield was greater following both 135 and 202 kg ha^−1^ of N compared with yield following 68 kg ha^−1^ of N. At Rocky Mount during that year, applying N at 202 kg ha^−1^ increased corn yield over the other lower rates of N. While no difference in corn yield was observed at Clayton during 2012 when comparing N rates, at Rocky Mount with each increase in N rate an increase in yield was observed. When comparing across N sources, no consistent trend was noted when comparing corn response to N rate.

Corn grain yield is often responsive to N rate, although response can vary based on soil and environmental conditions [21–23]. While corn response to N source and N rate was of interest in this research, the major focus was to address interactions of N source and N rate with timing of weed removal. Results from the current study suggest that corn response to UAN, SCU, and CPL at three N rates was not impacted by timing of weed removal within the first 28 days after planting. Although response to N rate and N source was variable, the lack of interactions of these treatment factors with weed height at timing of weed removal, reflecting the duration of weed interference, strengthens the argument that corn grain yield response to N fertilizer will not be impacted by timing of weed removal early in the season on soils typical to the North Carolina coastal plain.

## 4. Conclusions

Results from these experiments indicate that relationships between timing of weed removal and approaches to N management in corn exist with respect to N content in corn tissue early in the season with some of the applied N for corn being absorbed by weeds. Results also underscore the value in protecting corn grain yield by minimizing the length of time weeds interfere with corn by removing weeds with herbicides or with other approaches in different circumstances. However, even though some differences in N content in corn were noted early in the season due to weed interference, these differences did not translate into differences in corn grain yield. Considerable variation in corn grain yield response to N sources and N rates was noted across years and locations, and lack of an interaction of N source and rate with timing of weed removal strengthens the argument that corn will respond independently to N fertilization and early season weed interference on coastal plain soils in North Carolina. A reduction in grain yield due to increased duration of weed interference irrespective of N source or N rate is a reminder that timely weed control is critical regardless of fertility practices in corn.

## Figures and Tables

**Table 1:** Large crabgrass density as influenced by year, location, N source, and average height of weeds at timing of removal^a^.

Nitrogen source	Average weed height at timing of weed removal (cm)	2011		2012	
		Clayton	Rocky Mount	Clayton	Rocky Mount
		Number/0.25/m^2^			
Urea ammonium nitrate	8	89^a^	27^b^	104^b^	85^a^
Urea ammonium nitrate	16	36^b^	19^b^	87^b^	2^b^
Sulfur-coated urea	8	82^a^	37^a^	244^a^	119^a^
Sulfur-coated urea	16	47^b^	26^b^	68^b^	1^b^
Composted poultry litter	8	71^a^	39^a^	250^a^	66^a^
Composted poultry litter	16	50^b^	32^ab^	64^b^	2^b^

^a^Means within a year and location combination followed by the same letter are not significant according Fisher's protected LSD test at *p* ≤ 0.05. Data are pooled over N rates.

**Table 2:** Palmer amaranth density as impacted by the interaction of environment and N source and environment and average weed height at timing of weed removal^a^.

Treatment factor	2011		2012	
	Clayton	Rocky Mount	Clayton	Rocky Mount
	Number/0.25 m^2^			
*Nitrogen source* ^b^				
Urea ammonium nitrate	70^b^	18^a^	96^a^	1^a^
Sulfur-coated urea	57^b^	21^a^	156^a^	0^a^
Composted poultry litter	104^a^	17^a^	157^a^	0^a^
*Average weed height at timing of removal (cm)* ^c^				
8	100^a^	19^a^	95^a^	0^a^
16	55^b^	18^a^	85^a^	0^a^

^a^Means within a treatment factor and combination of location and year followed by the same letter are not significantly different according to Fisher's protected LSD test at *p* ≤ 0.05.

^b^Data are pooled over N rates and average weed height at timing of removal.

^c^Data are pooled over N sources and N rates.

**Table 3:** Nitrogen content in large crabgrass plants as influenced by the interaction of year, location, N source, N rate, and average weed height at timing of weed removal^a^.

		Nitrogen concentration in large crabgrass plants							
		Average weed height at timing of removal (cm)							
Nitrogen		2011				2012			
		Clayton		Rocky Mount		Clayton		Rocky Mount	
Source	Rate	8	16	8	16	8	16	8	16
	kg ha^−1^	kg ha^−1^							
Urea ammonium nitrate	68	5.5^c^	9.7^c^	2.5^b^	8.9^ab^	11.9^b^	7.5^b^	39.1^b^	0^b^
Urea ammonium nitrate	135	9.6^c^	12.3^c^	3.8^b^	5.9^b^	22.1^b^	28.1^b^	22.7^ab^	0.8^b^
Urea ammonium nitrate	202	7.0^c^	37.5^ab^	3.1^b^	3.5^b^	15.7^b^	34.0^ab^	10.3^b^	0^b^
Sulfur-coated urea	68	4.0^c^	8.6^c^	6.1^b^	9.7^ab^	16.1^b^	3.7^b^	13.7^b^	0^b^
Sulfur-coated urea	135	8.8^c^	32.0^b^	12.0^ab^	10.6^ab^	15.2^b^	8.6^b^	22.5^ab^	0^b^
Sulfur-coated urea	202	5.6^c^	51.7^b^	3.5^b^	5.2^b^	53.3^b^	11.9^b^	35.3^b^	0^b^
Composted poultry litter	68	4.3^c^	16.5^bc^	5.1^b^	14.9^b^	23.6^b^	6.8^b^	30.4^ab^	0.1^b^
Composted poultry litter	135	2.4^c^	27.2^bc^	5.2^b^	10.6^ab^	18.5^b^	20.3^b^	27.8^ab^	0^b^
Composted poultry litter	202	5.5^c^	16.2^bc^	11.8^ab^	7.7^ab^	13.7^b^	31.1^ab^	31.9^b^	0^b^

^a^Means within a year, location, and weed removal timing followed by the same letter are not significantly different at *p* ≤ 0.05.

**Table 4:** Nitrogen content in corn plants when weeds were removed at a height of 8 cm compared with weed-free corn as a result of the interaction of environment, N source, and N rate^a^.

		Nitrogen content in corn plants							
		Average weed height at timing of removal (cm)							
Nitrogen		2011				2012			
		Clayton		Rocky Mount		Clayton		Rocky Mount	
Source	Rate	0	8	0	8	0	8	0	8
	kg ha^−1^	kg ha^−1^							
Urea ammonium nitrate	68	2.3^c^	2.2^c^	0^c^	5.1^b^	2.3^a^	3.6^a^	3.8^a^	2.9^a^
Urea ammonium nitrate	135	6.1^ab^	3.2^bc^	10.0^ab^	5.2^b^	4.2^a^	4.6^a^	4.6^a^	4.4^a^
Urea ammonium nitrate	202	3.9^bc^	4.3^b^	8.5^ab^	5.4^b^	2.9^a^	2.6^a^	2.0^a^	3.9^a^
Sulfur-coated urea	68	3.7^bc^	2.2^c^	10.6^ab^	6.7^b^	3.6^a^	2.1^a^	2.4^a^	2.2^a^
Sulfur-coated urea	135	4.7^b^	2.8^bc^	4.0^bc^	5.8^b^	2.9^a^	4.2^a^	2.6^a^	3.3^a^
Sulfur-coated urea	202	7.8^a^	2.55^c^	12.5^a^	6.4^b^	1.7^a^	2.9^a^	4.5^a^	3.8^a^
Composted poultry litter	68	3.7^bc^	2.4^c^	8.7^ab^	4.9^bc^	2.9^a^	1.6^a^	4.0^a^	2.5^a^
Composted poultry litter	135	3.6^bc^	2.6^c^	9.7^ab^	5.2^b^	3.5^a^	4.1^a^	5.2^a^	4.3^a^
Composted poultry litter	202	3.7^bc^	2.5^c^	7.6^b^	5.5^b^	4.3^a^	4.0^a^	4.9^a^	4.2^a^

^a^Means within a year and location followed by the same letter are not significantly different according to Fisher's protected LSD test at *p* ≤ 0.05.

**Table 5:** Nitrogen content in corn plants when weeds were removed at a height of 16 cm compared with weed-free corn as a result of the interaction of environment, N source, and N rate^a^.

		Nitrogen content in corn plants							
		Average weed height at timing of removal (cm)							
Nitrogen		2011				2012			
		Clayton		Rocky Mount		Clayton		Rocky Mount	
Source	Rate	0	16	0	16	0	16	0	16
	kg ha^−1^	kg ha^−1^							
Urea ammonium nitrate	68	11.8^bcd^	7.0^de^	10.2^bcd^	9.7^cd^	6.6^a^	6.0^a^	5.8^a^	3.5^a^
Urea ammonium nitrate	135	19.3^ab^	3.5^e^	18.2^ab^	9.7^cd^	11.2^a^	10.0^a^	6.3^a^	4.1^a^
Urea ammonium nitrate	202	20.5^ab^	9.7^cd^	23.7^a^	11.3^bc^	11.1^a^	9.0^a^	2.7^a^	4.7^a^
Sulfur-coated urea	68	8.9^cde^	4.5^de^	—	7.4^cd^	9.4^a^	4.8^a^	3.2^a^	2.5^a^
Sulfur-coated urea	135	18.5^ab^	10.0^cd^	—	8.1^cd^	9.0^a^	10.9^a^	3.6^a^	3.4^a^
Sulfur-coated urea	202	27.7^a^	5.0^de^	21.3^ab^	12.4^bc^	6.5^a^	10.8^a^	6.0^a^	5.1^a^
Composted poultry litter	68	9.3^b–e^	4.2^de^	11.1^bcd^	7.1^de^	7.2^a^	5.6^a^	4.7^a^	3.7^a^
Composted poultry litter	135	16.4^bcd^	6.5^de^	11.5^bcd^	8.4^cd^	11.3^a^	7.7^a^	6.4^a^	4.9^a^
Composted poultry litter	202	16.6^b^	7.2^d^	16.2^bcd^	9.9^cd^	11.5^a^	10.1^a^	8.0^a^	5.2^a^

^a^Means within a year and location followed by the same letter are not significantly different according to Fisher's protected LSD test at *p* ≤ 0.05.

**Table 6:** Corn grain yield as influenced by the interaction of year, location, N source, and N rate^a^.

Nitrogen		2011		2012	
Source	Rate	Clayton	Rocky Mount	Clayton	Rocky Mount
	kg ha^−1^	kg ha^−1^			
Urea ammonium nitrate	68	2,270^b^	5,520^bc^	3,680^b^	2,940^bc^
Urea ammonium nitrate	135	2,730^ab^	7,480^ab^	4,710^ab^	4,680^ab^
Urea ammonium nitrate	202	3,490^ab^	6,900^b^	5,260^a^	4,560^ab^
Sulfur-coated urea	68	1,810^b^	5,180^c^	3,710^b^	2,050^c^
Sulfur-coated urea	135	3,850^a^	5,460^bc^	4,760^ab^	3,560^b^
Sulfur-coated urea	202	5,150^a^	8,640^a^	4,150^b^	5,350^a^
Composted poultry litter	68	3,480^ab^	5,090^c^	3,420^b^	2,810^bc^
Composted poultry litter	135	3,720^ab^	5,080^c^	3,510^b^	3,010^bc^
Composted poultry litter	202	3,440^ab^	5,380^bc^	4,230^b^	3,300^bc^

^a^Means within a combination of year and location followed by the same letter are not significantly different according to Fisher's protected LSD test at *p* < 0.05. Data are pooled over levels of average weed height at timing of removal.
